# Detection and Molecular Characterization of *Babesia canis vogeli* and *Theileria annulata* in Free-Ranging Dogs and Ticks from Shahriar County, Tehran Province, Iran

**DOI:** 10.18502/ijpa.v15i3.4196

**Published:** 2020

**Authors:** Gholamreza HABIBI, Alireza IMANI, Asghar AFSHARI, Soghra BOZORGI

**Affiliations:** 1. Department of Parasite Vaccine Research and Production, Razi Vaccine and Serum Research Institute, Agriculture Research, Education and Extension Organization (AREEO), Karaj, Iran; 2. Zhaweh Petclinic, Shahriar, Tehran, Iran

**Keywords:** *Babesia canis vogeli*, *Theileria annulata*, Polymerase chain reaction, Dog, Tick

## Abstract

**Background:**

We aimed to detect and characterize vector-borne parasites of *Babesia* and *Theileria* in dog and ticks by PCR assay. Canine babesiosis is a significant tick-borne disease caused by different *Babesia* species. As the infection has not been reported in Shahriar region Tehran, Iran, molecular techniques allowed us to identify tick-borne parasites in asymptomatic dogs.

**Methods:**

The number of 40 dog peripheral blood samples and 27 skin attached ticks were analyzed by molecular PCR assay. The specific primers were used for detecting *Babesia canis, B. gibsoni* and *T. annulata*.

**Results:**

*B. c. vogeli* was detected in 10 dog blood samples (25%). Additionally, *T. annulata* infection was identified in 13 dog blood samples (32.5%) and 18 isolated tick DNAs (66.7%). The results of PCR were confirmed by 18S rRNA and Tams1 gene sequence analyzing and have been registered in GenBank under following accession numbers for *B. c. vogeli* (MH793502) and *T. annulata* (MK105284). **Conclusion:** The verification of *T. annulata* infection in free-ranging dogs and ticks shows dogs might be considered as important natural carriers/reservoirs for *T. annulata* in enzootic region for bovine theileriosis. The obtained data may be useful for veterinary practitioners and dog owners to aware of *Babesia and Theileria* infection in dog and tick to establish the effective preventive measures.

## Introduction

Canine babesiosis is a tick-borne disease caused by the hemoprotozoa *Babesia canis* and *B. gibsoni* with world-wide distribution. The classic symptoms and signs of acute infection are characterized by hemolytic anemia, fever, hemoglobinuria, and may be lethal mainly in puppies (1).

*B. canis* is a large piroplasm (3.0–5.0 μm) and *B. gibsoni* is a small piroplasm (1.5–2.5 μm) during blood smear evaluation. There are three different subspecies of *B. canis*: *B. canis canis*, *B. canis rossi*, and *B. canis vogeli*. These subspecies are morphologically similar but had different vectors and pathogenicity (2).

Molecular methods, such as polymerase chain reaction (PCR), present a higher sensitivity and specificity than the peripheral blood smear evaluation to detect Babesial infection at the subspecies level differentiation (3).

With the advancement of molecular phylogenetic analysis, particularly 18S rRNA gene, it was recognized that *B. canis* has distinct subspecies, mainly *B. canis rossi*, *B. canis canis*, and *B. canis vogeli* (4–6). *B. canis vogeli* is found worldwide and is transmitted by *Rhipicephalus sanguineus* tick vector (7).

*B. canis vogeli* is the least virulent subspecies, but can cause clinical disease with severe anemia in puppies and subclinical infections with a low parasitemia in adult dogs (8, 9). *Theileria annulata* a protozoan parasite of cattle, but the strange occurrence of *T. annulata* infection has been recognized in dog in some parts of the world (10, 11).

*Theileria* species are intracellular protozoan parasites of wild and domestic ruminants transmitted by ixodid ticks. *T. annulata* is the causative agent of bovine theileriosis a disease of cattle widely distributed across southern Europe, North Africa and central Asia including Iran. *T. annulata* is transmitted by several *Hyalomma* tick species. *Theileria* infection causes a severe, and often fatal, disease of pure and cross-bred cattle. The tick vectors transmit *Theileria* infection from persistently infected cattle (carrier state) to a susceptible animal. Therefore, the existence of carrier animals is important for control and preventive program against theileriosis. The most probable reservoirs for *T. annulata* are ruminants like as cattle, sheep and camel, as well as wild ruminants, but recently some reports are for the role of canine from Europe, South Africa and Iran for *Theileria* infection in dogs (10–14).

Newly, we have pursued canine babesiosis in provided dog blood DNA samples with history of carrying tick on their surface bodies from Shahriar region, Tehran, Iran.

We aimed to examine free-ranging dog blood samples and their ticks for *Babesia* spp. and *T. annulata* infection by specific PCR assay.

## Materials and Methods

### Blood and tick samples

Peripheral EDTA-anticoagulated whole blood samples and skin attached ticks were taken from asymptomatic free-ranging owned dogs. Between Apr 2016 and Apr 2017, a total of 40 free-ranging owned dogs were examined for haemoprotozoan infection and tick infestations. Of these, 27 adult ticks were collected and preserved in 70% ethanol for morphological identification and DNA extraction.

The study was approved by local Ethics Committee.

### Morphological tick identification

Males *Rhipicephalus* tick species found on dogs could be differentiated by comparing the shape of adanal plates, accessory shields, and spiracular plates. Similarly, females of the same species could be distinguished based on genital opening, dorsal scutum, and spiracular plate shapes.

### Sampling region

**Fig. 1:**
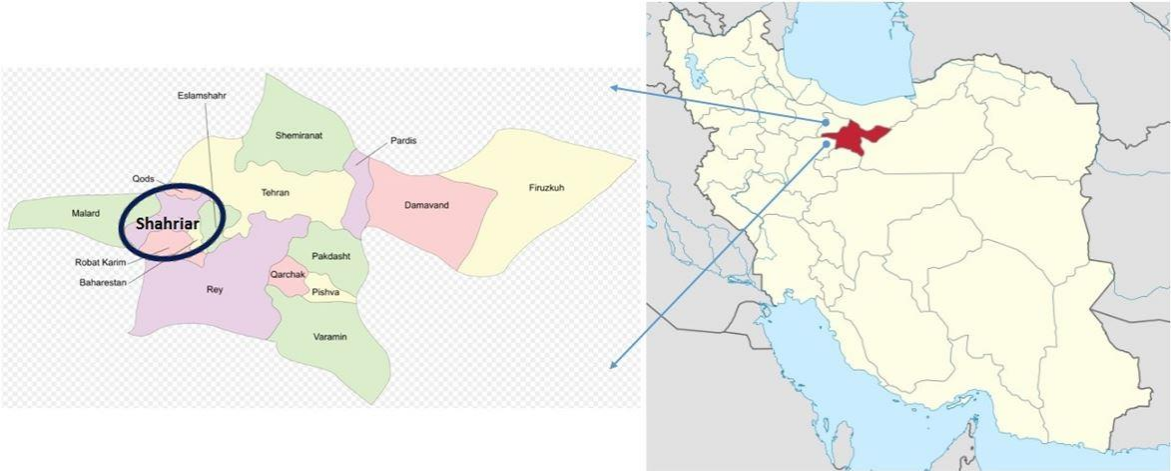
Location of Shahriar County in Tehran Province, Iran. This area has a large livestock population and numerous dairy cow farming, according to *T. annulata* infection the region is considered an enzootic district for bovine theileriosis (www.wikipedia.org)

Free-ranging owned dogs were brought to private Zhaweh petclinic in Shahriar County, Tehran Province, Iran (Fig. 1).

### DNA isolation

Proteinase K and further phenol chloroform purification were done for DNA extraction (15). Hard ticks were minced using liquid nitrogen, and then the tick lysate was subjected to lysis buffer and proteinase K treatment. The extracted DNA concentration was measured either by agarose gel electrophoresis and spectrophotometry (A_260_) and determining the ratio of A_260_/A_280_. Additionally, quality of the isolated DNA was estimated by agarose gel electrophoresis.

### PCR

Specific PCR assay for canine babesiosis was performed according to the Birkenheuer for detection and differentiation of three *B. canis* subspecies and *B. gibsoni* based on 18S rRNA gene sequence by semi-nested PCR technique (16) (Table 1). Specific PCR for *T. annulata* was performed based on the Tams1 gene sequence using a semi-nested PCR assay (Table 1).

**Table 1:** Oligonucleotide primers used for canine *Babesia* spp. and Theileria annulata amplification

***Primer***	***Sequence (5′-3′)***	***Reaction and/or use***
5–22F	GTTGATCCTGCCAGTAGT	Full-length 18S rRNA forward primer
1661R	AACCTTGTTACGACTTCTC	Full-length 18S rRNA reverse primer
455–479F	GTCTTGTAATTGGAATGATGGTGAC	Seminested PCR outer forward primer
793–772R	ATGCCCCCAACCGTTCCTATTA	Seminested PCR outer reverse primer
BgibAsia-F	ACTCGGCTACTTGCCTTGTC	Seminested PCR *B. gibsoni* specific forward primer
BCV-F	GTTCGAGTTTGCCATTCGTT	Seminested PCR *B. c. vogeli* specific forward primer
BCC-F	TGCGTTGACGGTTTGACC	Seminested PCR *B. c. canis* specific forward primer
BCR-F	GCTTGGCGGTTTGTTGC	Seminested PCR *B. c. rossi* specific forward primer
Tms92F	GAGACAAGGAATATTCTGAGTCC	Specific for *T. annulata* forward primer
Tms92R	TTAAGTGGCATATAATGACTTAAGC	Specific for *T. annulata* reverse primer
Tms92nF	CGGCACTGGAAAGAAGTACACC	Specific for *T. annulata* forward inner primer

* T. annulata PCR product for F and R primers is 597 and for semi-nested PCR by nF and R primers is 470 base pairs

PCR was performed in a final reaction volume of 20 μl containing 1X PCR premixed YektaTajhiz^TM^, 6 ul ddH_2_O, 10 pmol of each primers, and 2 μl of DNA template. The reactions were performed in an automatic DNA thermal cycler (Techne, Germany) with the first denaturation at 94 °C for 3 min and were followed by 35 cycles, each cycle consisted of a denaturing step of 10 seconds at 94 °C, an annealing step of 20 sec at (58 °C for *Babesia* spp*.* and 54 °C for *T. annulata*), and an extension step of 35 sec at 72 °C, followed by final extension step of 5 min at 72 °C.

### PCR product detection and sequencing

Amplified PCR products were separated by electrophoresis on 2% agarose gel, stained with RedSafe^TM^ (Nucleic Acid Staining Solution), and visualized by UV transillumination. PCR products were cleaned and extracted from agarose gel and were submitted for bidirectional DNA sequencing by using chain termination method (Takapouzist, Bioneer, South Korea).

### Blast analysis

The online program of blastn “Basic Local Alignment Search Tool (BLAST)” was used to find regions of local similarity between sequences by comparing the nucleotide or protein sequences to sequence databases and calculates the statistical significance of matches (https://blast.ncbi.nlm.nih.gov/). Two *B. canis vogeli* and *T. annulata* Shahriar isolates have sequenced and were analyzed using online blastn and finally registered in Gen-Bank.

### Phylogenetic analysis

The DNA sequences obtained from two studied *B. c. vogeli* and *T. annulata* samples and registered sequence databases in GenBank, were used for phylogenetic analysis by BLAST pairwise alignment. BLAST computes a pairwise alignment between a query and the database sequences searched. The evolutionary history was inferred using the Neighbor-Joining method (17). For purposes of this sequence tree presentation an implicit alignment between the database sequences is constructed, based upon the alignment of those (database) sequences to the query (Molecular Evolutionary Genetic Analysis, Ver. 6 [MEGA6]). All positions containing gaps and missing data were eliminated (18).

## Results

**Fig. 2:**
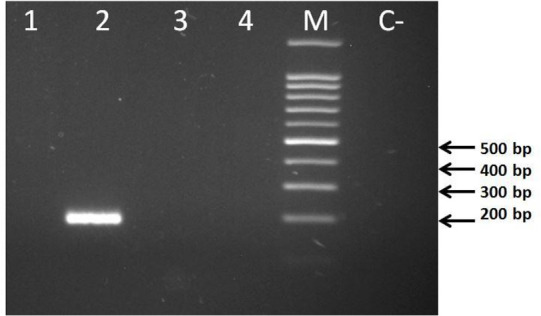
Gel electrophoresis for *Babesia canis vogeli* specific PCR on dog blood DNA. Lanes 1 to 4 are specific PCR for *B. gibsoni, B. c. vogeli* (positive sample, the expected size is 192 bp), *B. c. canis* and *B. c. rossi* respectively, M is 100 bp DNA size marker and C- is the negative control

**Fig. 3:**
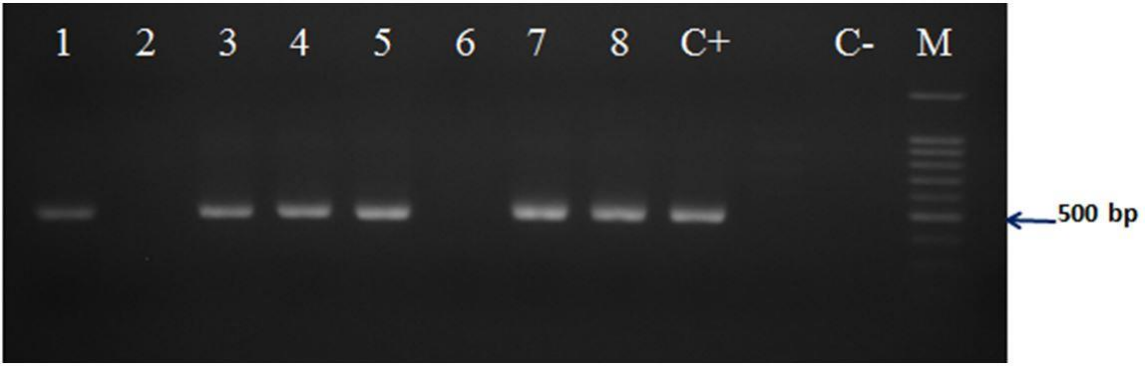
Gel electrophoresis for *Theileria annulata* specific seminested-PCR on dog blood DNAs. All samples except number 2 and 6 were shown positive as well as positive control (C+). C− is the negative control and M is 100 bp DNA size marker

The expected amplicons with the sizes of 192 bp for *B. c. vogeli* 18S rRNA gene, partial sequence and 470 bp for *T. annulata* Tams1 gene, partial sequence were observed in all of the examined positive samples (Fig. 2 and Fig. 3).

The PCR for *Babesia spp.* identification were performed by the specific primers for 18S rRNA gene sequences of *B. c. canis, B. c. rossi, B. c. vogeli and B. gibsoni*. The results have revealed no amplification for *B. c. canis, B. c. rossi* and *B. gibsoni*, but the specific amplification was seen in 10 of all 40 dog blood samples for *B. c. vogeli* subspecies (Fig. 2).

**Fig. 4:**
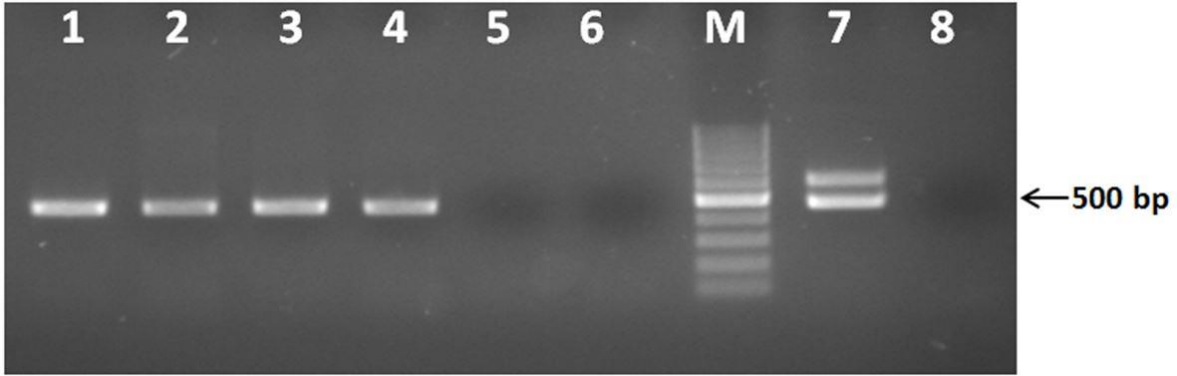
Gel electrophoresis for *T. annulata* specific PCR on tick DNAs. Lanes 1 to 6 are specific PCR for *T. annulata* on tick DNAs (samples 1 to 4 are positive), lane 7, *T. annulata* positive control, lane 8, negative control and M is 100 bp DNA size marker

The PCR for *T. annulata* by the specific primers for Tams1 gene sequence of *T. annulata* was determined that 13 of all 40 dog blood samples and 18 of all 27 *R. sanguineus* tick lysates were positive for *T. annulata* infection (Fig. 3 and Fig. 4).

The sequences of the 18S rRNA gene of the *B. c. vogeli* Shahriar isolate and Tams1 gene of *T. annulata* Shahriar isolate were verified by DNA sequencing and registered in GenBank under accession number of MH793502 and MK105284 respectively.

Results of nucleotide Blast and phylogenetic analysis for *B. c. vogeli.* The identity percent was determined by online Blastn program for comparison of the obtained *B. c. vogeli* 18S rRNA sequence Iran isolate and other registered sequences in GenBank. The Blast analysis has showed 99–100% identity between Shahriar isolate and other registered *B. c. vogeli* sequences from Turkey, Romania and Egypt. Moreover, MEGA6 program analysis verified the mentioned above results. The sequence of *B. c. vogeli* 18S rRNA Iran isolate shows a very high degree of similarity with the isolates from Egypt, Turkey, Tunisia and Romania (100% identical). The moderate degree of sequence divergence was seen between Iran isolate and sequences from Brazil, Peru, Paraguay, Zambia, China, Thailand and Portugal (0.006 to 0.010). But the highest level of diversity was estimated between *B. c. vogeli* Iran isolate and sequences from India and Russia (0.026, 0.049 respectively).

**Fig. 5:**
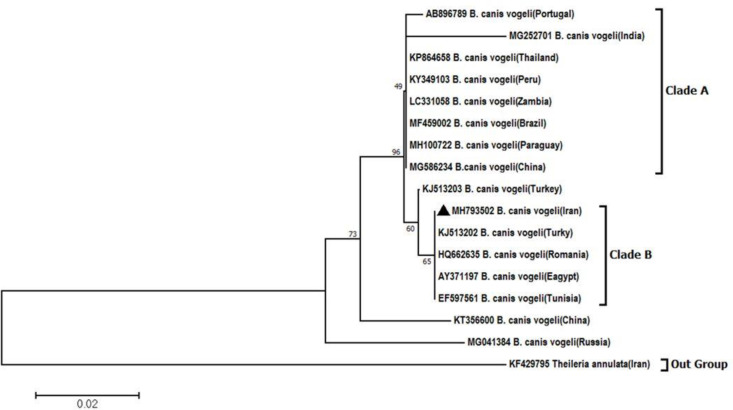
The rooted phylogenetic tree for *B. c. vogeli* Iran isolate and 16 different 18S rRNA gene sequences registered in GenBank. The sequences were grouped in two major clades (detailed described in the text). *Theileria annulata* as an outgroup sequence is the least related to the group of taxa that we are studying). Scale bar represents nucleotide substitutions per position

The phylogenetic tree was constructed based on the 18S rRNA gene sequence for *B. c. vogeli* Iran isolate and registered sequences in Gen-Bank. The phylogenetic analysis was performed using Neighbor-Joining method depicted in a rooted tree. The analysis involved 17 *B. c. vogeli* 18S rRNA nucleotide sequences. There were total of 309 positions in the final dataset. Evolutionary analyses were conducted in MEGA6 program. The *B. c. vogeli* 18S rRNA gene sequences were divided in two main clades and *B. c. vogeli* Iran isolate was close to isolates from Turkey, Romania, Egypt and Tunisia in clade B. However, *B. c. vogeli* isolates from China, Brazil, Zambia, Peru, Thailand, India, Paraguay and Portugal were in clade A (Fig. 5).

Results of nucleotide Blast and Phylogenetic analysis for *Theileria annulata.* The identity percent was determined by online blastn program for comparison of the obtained *T. annulata* Tams1 sequence of dog isolate (MK105284) and other registered sequences in GenBank. The homology was shown up to 98 percent between *T. annulata* Iran dog isolate and other *T. annulata* Tams1 sequences registered in GenBank.

The phylogenetic tree was constructed based on the Tams1 gene sequence of *T. annulata* for Shahriar dog isolate and registered Tams1 sequences in GenBank. The phylogenetic analysis was performed using Neighbor-Joining method depicted in an unrooted tree. The analysis involved 17 *T. annulata* Tams1 nucleotide sequences.

**Fig. 6:**
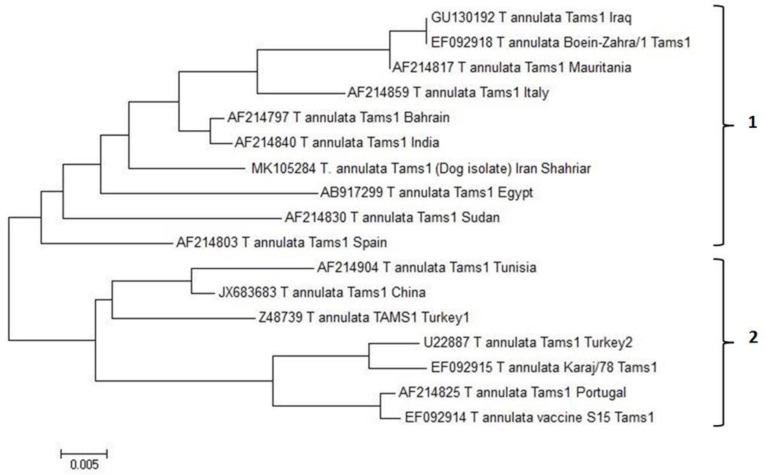
Phylogenetic analysis of *T. annulata* based on Tams1 gene sequences. The analysis involved of 17 *T. annulata* Tams1 gene sequences including Shahriar dog isolate. The tree is drawn to scale, with branch lengths in the same units as those of the evolutionary distances used to infer the phylogenetic tree

Evolutionary analyses were conducted in MEGA6. Tams1 gene sequences were divided in two main clades and *T. annulata* dog isolate was close to *T. annulata* isolates from India and Bahrain, Italy, Egypt, Sudan, Spain, Mauritania, Iraq and Iran (Boein Zahra isolate) in the first clade. The second clade consists of *T. annulata* isolates from Tunisia, China, Turkey, Portugal and Iran (Fig. 6).

**Fig. 7:**
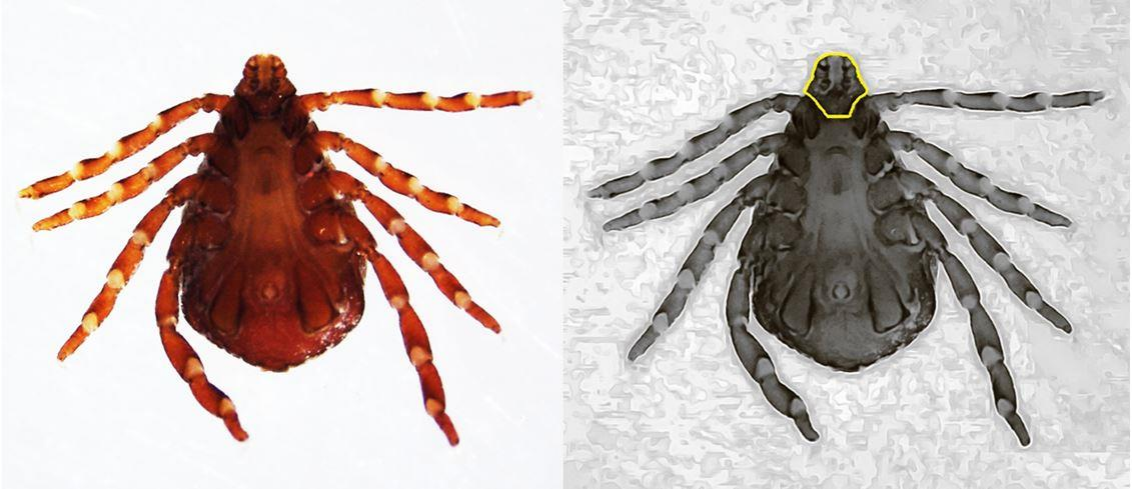
*Rhipicephalus sanguineus* adult male tick infected with *Theileria annulata* from Shahriar County. The adult brown dog tick (*R. sanguineus*) is small and red brown in color and no ornamentation on the body. It can be recognized by its elongated body and hexagonal capituli. Hexagonal basis capituli, an identifying character for the brown dog tick, *R. sanguineus* (shown in the right picture)

The results of morphological inspection of ticks. As mentioned earlier, the number of 27 ticks were collected and studied for identification of Piroplasmida. All ticks were examined and according to the key criteria were identified as *Rhipicephalus sanguineus* (brown dog tick) (Fig. 7).

## Discussion

Tick-borne piroplasms consist of two important protozoan genera *Theileria* and *Babesia*. Canine babesiosis is a significant tick-borne disease caused by different *Babesia* species. As the infection has not been reported in Shahriar region, molecular techniques allowed us to identify of tick-borne parasites in asymptomatic free-ranging owned dogs and ticks. First, the aim of this study was to identify *Babesia* spp., but according to the reports on the presence of *Theileria* spp. infection in dogs, the investigation was extended for detecting *T. annulata*. The results of the present study revealed a significant number of samples have infected to *Babesia* and *Theileria* in dog blood samples and ticks.

The specific PCR primers were used for detection of canine babesiosis for 18S rRNA gene, based on the nested-PCR and semi-nested PCR by four specific primers for differentiation of three *B. canis* sub species; *B. c. canis, B. c. rossi* and *B. c. vogeli* and *B. gibsoni* as well. The results showed us 25% of dog blood samples were infected to *B. c. vogeli* and the obtained data of DNA sequencing has recon-firmed the result.

Generally, *R. sanguineus* is often collected from domestic ruminants. Nevertheless, *R. sanguineus* is considered an important vector to transmit many bacterial and parasitic agents, including *B. vogeli*, *Ehrlichia canis*, *Mycoplasma haemocanis*, *Hepatozoon canis* to carnivores (19, 20).

In this study the number of 27 ticks was collected in Shahriar county of Tehran Province, and all were diagnosed as *R. sanguineus,* in agreement with previous reports performed in Iran reported from different geographic areas; *R. sanguineus* in Isfahan Province (6.5% in cattle), *Rhipicephalus* spp. in West and North-West of Iran (in transmission of bovine *Babesia* spp.), *R. sanguineus* in west parts of Iran (62% in small ruminants), *R. sanguineus* in Alashtar of Iran (17% in cattle) and *R. sanguineus* in Northern of Iran (82.35% of small ruminants), *R. sanguineus* in Southeast of Iran (21–26).

The obtained results of this study for *B. c. vogeli* infection in 10 of 40 dogs blood samples (25%) in Shahriar region confirmed previous studies; *B. canis* was reported in one splenectomized dog in north of Iran (27), *B. canis* DNA were detected by PCR from 9 (7.5%) out of 120 dogs in Chaharmahal Va Bakhtiari provinces of Iran (28), *B. canis* was detected among 400 dogs (3.75%) in Ahwaz distric of Iran (29).

According to the previous unusual reports for *T. annulata* infection in free-ranging dogs from Spain and Southern Iran, thus we examined the dog blood samples and ticks for *T. annulata* infection. In this study the DNA sequences for *T. annulata* were found in 13 samples of 40 dog blood samples (32.5%) as well as 18 tick lysates of 27 ticks (66.7%) in Shahriar County of Tehran Province. These findings are in accordance with previous studies; *T. annulata* infection in dog from Shiraz (South of Iran) was detected by PCR (11), *T. annulata* infection in dog from Spain (10), *Theileria* spp., and *T. equi* infection in dog from South Africa (14), and *T. annae* infection in dog from Spain and Sweden (12, 13).

However, based on the recent reports, it seems that other hosts like as dogs could be a natural carrier for *Theileria* spp. infection, including *T. annulata*. *Hyalomma* spp. group are considered as main vector for *Theileria* transmission but according to the above mentioned reports, *R. sanguineusis* the brown dog tick with a vast worldwide distribution may be have an important role in *Theileria spp*. transmission.

Although, the blood smear examination by microscopy considered a routine and applied method for detection of babesiosis, but its sensitivity is less than molecular methods to finding a precise diagnosis (19), Moreover, *B. c. vogeli* could not easily be identified by microscopic examination (30). For this reason, more sensitive molecular methods like as PCR-based methods, are recommended (31).

The clinical symptoms of canine babesiosis are different, from subclinical infections to involvement of various organs and even fatal risk. It should be noted that many carrier dogs that have chronic infection, will not show clinical signs, due to premunition, unless there is a major problem with animal health (32).

PCR is a useful screening tool in dogs that are constantly exposed to piroplasms. The chronic condition of infection in these dogs makes them susceptible for illness relapse or continuing the chronic infection. In these situations, the PCR can be used appropriately to show whether the infection remains or has been likely removed (33).

The most important way to prevent canine babesiosis is tick control. Vertical transmission of *Babesia spp.* in tick vectors indicates that the tick population in the area has been able to remain infected for a long time and the dogs in that area are re-infected, and this progressive increasing of infected ticks will be repeated. The preventive measures consist of regular monitoring for ticks by pet owners and veterinarians, as well as the use of acaricidal treatment (34).

Additionally, a vaccine is available to prevent babesiosis in dogs. This vaccine is designed to protect dogs against *B. canis* called Pirodog® (Merial). The vaccine contains parasite-soluble antigens isolated from culture medium and provides a partial protection for dogs that are newly exposed to *B. canis* infection, which reduces the severity of clinical symptoms. However, vaccination does not prevent infection, but it seems to stop many pathological processes involved in the disease (35).

## Conclusion

This study is the first molecular detection and characterization of *B canis vogeli* from free ranging dogs in Shahriar County by specific PCR method. Moreover, co-infection of *T. annulata* as a ruminant pathogen was detected and characterized in dog blood samples and *R. sanguineus* tick vectors by PCR. This asymptomatic state of *B. c. vogeli* and *T. annulata* infected dog as well as *T. annulata* infection in *R. sanguineus* tick vector might be important for better understanding the epizoology of canine babesiosis and bovine theileriosis in enzootic region and potential role of infected dog for natural reservoir to continuing *T. annulata* life cycle.

## Ethical considerations

Ethical issues (Including plagiarism, informed consent, misconduct, data fabrication and/or falsification, double publication and/or submission, redundancy, etc.) have been completely observed by the authors.
